# Integrating adolescent substance abuse screening, brief intervention and treatment in health professions education

**DOI:** 10.1186/1940-0640-10-S2-O36

**Published:** 2015-09-24

**Authors:** Tracy McPherson, Eric Goplerud, Cyrille Adam

**Affiliations:** 1 NORC at the University of Chicago, Chicago, USA; 2 Kognito, New York City, USA

## Background

Substance abuse remains one of the largest public health issues facing our society. Early alcohol and drug use is linked to a range of immediate and long-term consequences (e.g., academic, brain development, and later dependence). Although studies such as the Youth Risk Behavior Survey and National Survey on Drug Use and Health have recently shown stable or slight decline in the use of alcohol and certain drugs, alcohol remains the drug of choice, marijuana use has increased, and perceptions of harm has decreased.[1,2] Opportunities to address substance use exist in a range of settings where nurses, social workers and other health professionals work with youth yet training and adoption of adolescent screening and brief intervention has been slow. The cost of substance use disorders, in both adults and adolescents, could be reduced through the SAMHSA-backed prevention and early identification procedure of Screening, Brief Intervention, and Referral to Treatment (SBIRT), a cost-effective and widely-supported prevention framework. Research has shown SBIRT to be effective for the early identification of problematic alcohol use, with growing but inconsistent evidence for its effectiveness with other risky drug use. However, there is little substance use training in social work and nursing programs. SBIRT education is often optional or specialized, rather than being required coursework.

## Material and methods

NORC at the University of Chicago has been funded by the Conrad N. Hilton Foundation to increase training opportunities in adolescent SBIRT within undergraduate and graduate social work and nursing programs. In October 2014, NORC partnered with the Council on Social Work Education, Center for Clinical Social Work, American Association of Colleges of Nursing, and New York-based technology company Kognito to support the integration of adolescent SBIRT education into required coursework for their students.

Following the model established by the Institute for Healthcare Improvement,[3] NORC has assembled a Learning Collaborative comprised of faculty from schools of nursing and social work throughout the United States. Led by a Steering Committee including partnering organizations and experts on adolescent substance use, the Learning Collaborative has been developing curricular and technical assistance tools to integrate substance use education in nursing and social work education.

## Results

To date, faculty from approximately 100 schools of nursing and social work have actively contributed to the initiative. Tools created include a virtual patient simulation that will allow students to learn specifics of SBIRT and the delivery of tailored brief interventions based on motivational interviewing.[4] Developed by Kognito, the online simulation enables learners to practice brief interventions in conversation with realistic virtual patients, and facilitates assessment of competency by scoring learners’ performance and providing elaborative feedback.

## Conclusions

There is wide-ranging support among schools of social work and nursing for the integration of SBIRT education in curricula with support from professional associations and technical assistance providers. Further study underway will evaluate the intitative’s effectiveness in developing substance use competencies among health professionals.
